# (2*E*)-2-(4-Meth­oxy­benzyl­idene)-2,3-di­hydro-1*H*-inden-1-one

**DOI:** 10.1107/S1600536812006940

**Published:** 2012-02-24

**Authors:** Abdullah M. Asiri, Hassan M. Faidallah, Khulud F. Al-Nemari, Seik Weng Ng, Edward R. T. Tiekink

**Affiliations:** a Center of Excellence for Advanced Materials Research, King Abdulaziz University, PO Box 80203, Jeddah 21589, Saudi Arabia; b Chemistry Department, Faculty of Science, King Abdulaziz University, PO Box 80203, Jeddah 21589, Saudi Arabia; c Department of Chemistry, University of Malaya, 50603 Kuala Lumpur, Malaysia

## Abstract

In the title compound, C_17_H_14_O_2_, the indan-1-one system is almost planar (r.m.s. deviation = 0.007 Å) and the benzene ring is twisted out of its plane by 8.15 (6)°. The conformation about the C=C double bond [1.348 (2) Å] is *E*. Helical supra­molecular chains along [010] feature in the crystal packing; these are sustained by C—H⋯O hydrogen bonds and π–π inter­actions between translationally related indan-1-one systems [centroid–centroid distance = 3.7970 (10) Å].

## Related literature

For related cyclic ketone structures, see: Asiri, Faidallah & Ng (2011 ▶); Asiri, Al-Youbi *et al.* (2011 ▶).
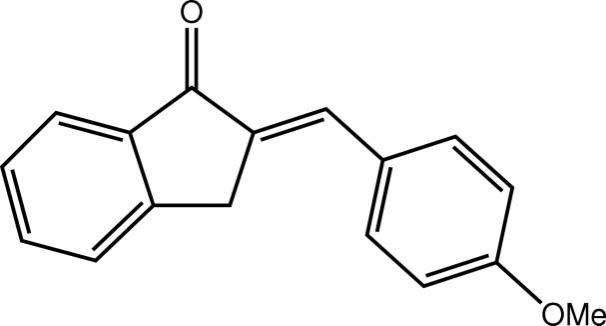


## Experimental

### 

#### Crystal data

C_17_H_14_O_2_
*M* *_r_* = 250.28
Monoclinic, 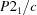

*a* = 15.1177 (10) Å
*b* = 3.9322 (3) Å
*c* = 20.7072 (13) Å
β = 94.615 (6)°
*V* = 1226.97 (15) Å^3^
*Z* = 4
Mo *K*α radiation
μ = 0.09 mm^−1^
*T* = 100 K
0.30 × 0.30 × 0.03 mm

#### Data collection

Agilent SuperNova Dual diffractometer with an Atlas detector
Absorption correction: multi-scan (*CrysAlis PRO*; Agilent, 2011 ▶) *T*_min_ = 0.974, *T*_max_ = 0.997
4873 measured reflections
2792 independent reflections
2131 reflections with *I* > 2σ(*I*)
*R*_int_ = 0.030

#### Refinement

*R*[*F* ^2^ > 2σ(*F* ^2^)] = 0.049
*wR*(*F* ^2^) = 0.126
*S* = 1.03
2792 reflections
172 parameters
H-atom parameters constrained
Δρ_max_ = 0.24 e Å^−3^
Δρ_min_ = −0.25 e Å^−3^

### 

Data collection: *CrysAlis PRO* (Agilent, 2011 ▶); cell refinement: *CrysAlis PRO*; data reduction: *CrysAlis PRO*; program(s) used to solve structure: *SHELXS97* (Sheldrick, 2008 ▶); program(s) used to refine structure: *SHELXL97* (Sheldrick, 2008 ▶); molecular graphics: *X-SEED* (Barbour, 2001 ▶) and *DIAMOND* (Brandenburg, 2006 ▶); software used to prepare material for publication: *publCIF* (Westrip, 2010 ▶).

## Supplementary Material

Crystal structure: contains datablock(s) global, I. DOI: 10.1107/S1600536812006940/hb6631sup1.cif

Structure factors: contains datablock(s) I. DOI: 10.1107/S1600536812006940/hb6631Isup2.hkl

Supplementary material file. DOI: 10.1107/S1600536812006940/hb6631Isup3.cml

Additional supplementary materials:  crystallographic information; 3D view; checkCIF report

## Figures and Tables

**Table 1:** Hydrogen-bond geometry (Å, °)

*D*—H⋯*A*	*D*—H	H⋯*A*	*D*⋯*A*	*D*—H⋯*A*
C13—H13⋯O1^i^	0.95	2.58	3.5327 (19)	175

Symmetry code: (i) 
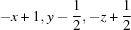
.

**Fig. 1.**
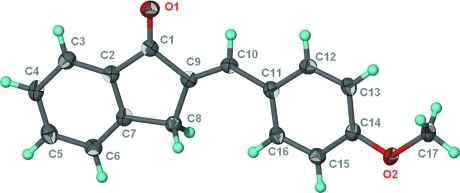
The molecular structure of (I) showing displacement ellipsoids at the 70% probability level.

**Fig. 2.**
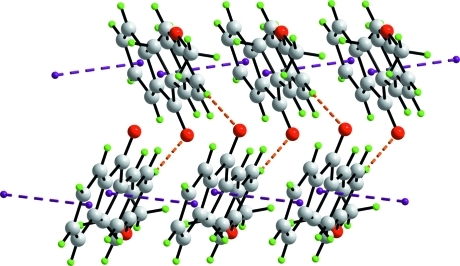
A view of the helical supramolecular chain along [010] in (I). The C—H···O and π–π interactions are shown as orange and purple dashed lines, respectively.

**Fig. 3.**
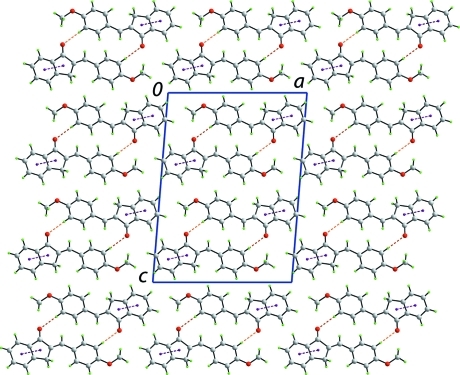
A view in projection down the b axis of the unit-cell contents for (I), highlighting the stacking of chains.

## supplementary crystallographic information

### Comment

The title compound, 2-(4-methoxybenzylidene)indan-1-one (I), was investigated in connection with recent structure determinations of related cyclic ketone derivatives (Asiri, Faidallah & Ng, 2011; Asiri, Al-Youbi *et al.*, 2011).

The nine non-hydrogen atoms of the inden-1-one system in (I), Fig. 1, are co-planar with a r.m.s. deviation = 0.007 Å. The dihedral angle between the inden-1-one system and benzene ring is 8.15 (6)°, and the methoxy substituent is co-planar with the benzene ring to which it is connected [the C17—O2—C14—C13 torsion angle = -0.6 (2)°]. The configuration about the C9═C10 double bond [1.348 (2) Å] is *E*.

In the crystal packing, molecules aggregate along the 2_1_ axis *via* C—H···O, Table 1, and π(C1,C2,C7—C9)···π(C2–C7)^i^ interactions between symmetry related rings of the inden-1-one system [centroid···centroid distance = 3.7970 (10) °, angle between rings = 0.51 (8)° for *i*: *x*, -1 + *y*, *z*]. There are no specific interactions between the supramolecular chains, Fig. 3.

### Experimental

A solution of the *p*-anisaldehyde (1.36 g, 0.01 mol) in ethanol (20 ml) was added to a stirred solution of 1-indanone (1.3 g,0.01 mol) in ethanolic KOH (20%, 20 ml), and stirring was maintained at room temperature for 6 h. The reaction mixture was then poured onto water (200 ml) and set aside overnight. The precipitated solid product was collected by filtration, washed with water, dried and recrystallized from its ethanol solution as light-brown plates, *M*.pt.: 491–493 K.

### Refinement

Carbon-bound H-atoms were placed in calculated positions [C—H = 0.95 to 0.99 Å, *U*_iso_(H) = 1.2*U*_eq_(C)] and were included in the refinement in the riding model approximation. Two reflections, *i.e*. (1 0 2) and (0 0 14), were omitted owing to poor agreement.

### Crystal data

**Table:**

C_17_H_14_O_2_	*F*(000) = 528
*M**_r_* = 250.28	*D*_x_ = 1.355 Mg m^−^^3^
Monoclinic, *P*2_1_/*c*	Mo *K*α radiation, λ = 0.71073 Å
Hall symbol: -P 2ybc	Cell parameters from 1476 reflections
*a* = 15.1177 (10) Å	θ = 2.3–27.5°
*b* = 3.9322 (3) Å	µ = 0.09 mm^−^^1^
*c* = 20.7072 (13) Å	*T* = 100 K
β = 94.615 (6)°	Plate, light brown
*V* = 1226.97 (15) Å^3^	0.30 × 0.30 × 0.03 mm
*Z* = 4	

### Data collection

**Table:**

Agilent SuperNova Dual diffractometer with an Atlas detector	2792 independent reflections
Radiation source: SuperNova (Mo) X-ray Source	2131 reflections with *I* > 2σ(*I*)
Mirror monochromator	*R*_int_ = 0.030
Detector resolution: 10.4041 pixels mm^-1^	θ_max_ = 27.6°, θ_min_ = 2.7°
ω scan	*h* = −14→19
Absorption correction: multi-scan (*CrysAlis PRO*; Agilent, 2011)	*k* = −3→5
*T*_min_ = 0.974, *T*_max_ = 0.997	*l* = −26→22
4873 measured reflections	

### Refinement

**Table:**

Refinement on *F*^2^	Primary atom site location: structure-invariant direct methods
Least-squares matrix: full	Secondary atom site location: difference Fourier map
*R*[*F*^2^ > 2σ(*F*^2^)] = 0.049	Hydrogen site location: inferred from neighbouring sites
*wR*(*F*^2^) = 0.126	H-atom parameters constrained
*S* = 1.03	*w* = 1/[σ^2^(*F*_o_^2^) + (0.054*P*)^2^ + 0.3168*P*] where *P* = (*F*_o_^2^ + 2*F*_c_^2^)/3
2792 reflections	(Δ/σ)_max_ = 0.001
172 parameters	Δρ_max_ = 0.24 e Å^−^^3^
0 restraints	Δρ_min_ = −0.25 e Å^−^^3^

### Fractional atomic coordinates and isotropic or equivalent isotropic displacement parameters (Å^2^)

**Table:**

	*x*	*y*	*z*	*U*_iso_*/*U*_eq_	
O1	0.21241 (7)	0.4870 (3)	0.24618 (5)	0.0249 (3)	
O2	0.73625 (7)	0.5812 (3)	0.43081 (5)	0.0233 (3)	
C1	0.22115 (10)	0.6064 (4)	0.30095 (8)	0.0186 (4)	
C2	0.15106 (10)	0.7692 (4)	0.33660 (7)	0.0184 (4)	
C3	0.06204 (10)	0.8185 (4)	0.31668 (8)	0.0216 (4)	
H3	0.0384	0.7449	0.2751	0.026*	
C4	0.00833 (11)	0.9776 (4)	0.35877 (8)	0.0227 (4)	
H4	−0.0526	1.0155	0.3460	0.027*	
C5	0.04381 (11)	1.0821 (5)	0.42005 (8)	0.0240 (4)	
H5	0.0063	1.1894	0.4486	0.029*	
C6	0.13295 (11)	1.0321 (4)	0.44003 (8)	0.0215 (4)	
H6	0.1565	1.1042	0.4817	0.026*	
C7	0.18690 (10)	0.8739 (4)	0.39758 (8)	0.0187 (4)	
C8	0.28497 (10)	0.7948 (4)	0.40744 (7)	0.0193 (4)	
H8A	0.2978	0.6405	0.4448	0.023*	
H8B	0.3203	1.0054	0.4146	0.023*	
C9	0.30473 (10)	0.6238 (4)	0.34465 (7)	0.0181 (4)	
C10	0.38144 (10)	0.5031 (4)	0.32473 (7)	0.0182 (4)	
H10	0.3758	0.3929	0.2838	0.022*	
C11	0.47172 (10)	0.5132 (4)	0.35550 (7)	0.0183 (4)	
C12	0.53982 (10)	0.3750 (4)	0.32146 (8)	0.0193 (4)	
H12	0.5244	0.2673	0.2811	0.023*	
C13	0.62868 (10)	0.3885 (4)	0.34408 (8)	0.0200 (4)	
H13	0.6731	0.2933	0.3196	0.024*	
C14	0.65108 (10)	0.5448 (4)	0.40343 (8)	0.0189 (4)	
C15	0.58486 (10)	0.6782 (4)	0.43933 (8)	0.0207 (4)	
H15	0.6005	0.7811	0.4802	0.025*	
C16	0.49691 (10)	0.6619 (4)	0.41602 (7)	0.0197 (4)	
H16	0.4526	0.7524	0.4412	0.024*	
C17	0.80559 (10)	0.4433 (5)	0.39500 (8)	0.0242 (4)	
H17	0.8629	0.4734	0.4200	0.036*	
H17B	0.8063	0.5621	0.3534	0.036*	
H17C	0.7948	0.2005	0.3872	0.036*	

### Atomic displacement parameters (Å^2^)

**Table:**

	*U*^11^	*U*^22^	*U*^33^	*U*^12^	*U*^13^	*U*^23^
O1	0.0235 (6)	0.0315 (7)	0.0198 (6)	−0.0012 (5)	0.0025 (5)	−0.0041 (5)
O2	0.0152 (6)	0.0311 (7)	0.0235 (6)	0.0017 (5)	−0.0001 (4)	−0.0045 (5)
C1	0.0193 (8)	0.0181 (8)	0.0187 (8)	−0.0016 (7)	0.0037 (6)	0.0015 (7)
C2	0.0189 (8)	0.0186 (9)	0.0181 (8)	−0.0012 (7)	0.0033 (6)	0.0029 (7)
C3	0.0200 (8)	0.0225 (9)	0.0221 (8)	−0.0033 (7)	0.0009 (6)	0.0017 (7)
C4	0.0171 (8)	0.0248 (9)	0.0263 (8)	0.0013 (7)	0.0023 (6)	0.0050 (8)
C5	0.0221 (8)	0.0261 (9)	0.0247 (9)	0.0028 (8)	0.0077 (7)	0.0027 (8)
C6	0.0235 (8)	0.0215 (9)	0.0195 (8)	0.0010 (7)	0.0023 (6)	0.0006 (7)
C7	0.0189 (8)	0.0169 (8)	0.0202 (8)	−0.0006 (7)	0.0022 (6)	0.0035 (7)
C8	0.0188 (8)	0.0207 (9)	0.0183 (8)	0.0001 (7)	0.0010 (6)	−0.0001 (7)
C9	0.0196 (8)	0.0167 (8)	0.0183 (8)	−0.0011 (7)	0.0029 (6)	0.0015 (7)
C10	0.0217 (8)	0.0174 (8)	0.0158 (7)	−0.0017 (7)	0.0029 (6)	0.0007 (7)
C11	0.0192 (8)	0.0166 (8)	0.0192 (7)	0.0008 (7)	0.0032 (6)	0.0028 (7)
C12	0.0224 (8)	0.0188 (8)	0.0169 (7)	−0.0004 (7)	0.0019 (6)	−0.0008 (7)
C13	0.0189 (8)	0.0214 (9)	0.0203 (8)	0.0028 (7)	0.0051 (6)	0.0013 (7)
C14	0.0168 (8)	0.0196 (8)	0.0202 (8)	0.0003 (7)	0.0008 (6)	0.0034 (7)
C15	0.0237 (8)	0.0222 (9)	0.0160 (7)	0.0029 (7)	0.0014 (6)	−0.0005 (7)
C16	0.0193 (8)	0.0215 (9)	0.0187 (8)	0.0031 (7)	0.0046 (6)	0.0013 (7)
C17	0.0149 (8)	0.0292 (10)	0.0285 (9)	0.0025 (7)	0.0022 (6)	−0.0025 (8)

### Geometric parameters (Å, º)

**Table:**

O1—C1	1.2249 (19)	C8—H8B	0.9900
O2—C14	1.3721 (19)	C9—C10	1.348 (2)
O2—C17	1.4379 (18)	C10—C11	1.460 (2)
C1—C2	1.484 (2)	C10—H10	0.9500
C1—C9	1.495 (2)	C11—C12	1.403 (2)
C2—C3	1.389 (2)	C11—C16	1.407 (2)
C2—C7	1.396 (2)	C12—C13	1.388 (2)
C3—C4	1.387 (2)	C12—H12	0.9500
C3—H3	0.9500	C13—C14	1.391 (2)
C4—C5	1.399 (2)	C13—H13	0.9500
C4—H4	0.9500	C14—C15	1.396 (2)
C5—C6	1.392 (2)	C15—C16	1.379 (2)
C5—H5	0.9500	C15—H15	0.9500
C6—C7	1.393 (2)	C16—H16	0.9500
C6—H6	0.9500	C17—H17	0.9800
C7—C8	1.512 (2)	C17—H17B	0.9800
C8—C9	1.515 (2)	C17—H17C	0.9800
C8—H8A	0.9900		
			
C14—O2—C17	116.51 (12)	C10—C9—C8	130.76 (14)
O1—C1—C2	126.71 (14)	C1—C9—C8	108.93 (13)
O1—C1—C9	126.93 (14)	C9—C10—C11	130.85 (15)
C2—C1—C9	106.35 (13)	C9—C10—H10	114.6
C3—C2—C7	121.58 (15)	C11—C10—H10	114.6
C3—C2—C1	128.75 (15)	C12—C11—C16	116.83 (14)
C7—C2—C1	109.67 (14)	C12—C11—C10	117.92 (14)
C4—C3—C2	118.60 (15)	C16—C11—C10	125.20 (14)
C4—C3—H3	120.7	C13—C12—C11	123.04 (15)
C2—C3—H3	120.7	C13—C12—H12	118.5
C3—C4—C5	120.09 (15)	C11—C12—H12	118.5
C3—C4—H4	120.0	C12—C13—C14	118.37 (14)
C5—C4—H4	120.0	C12—C13—H13	120.8
C6—C5—C4	121.30 (15)	C14—C13—H13	120.8
C6—C5—H5	119.3	O2—C14—C13	124.38 (14)
C4—C5—H5	119.3	O2—C14—C15	115.46 (14)
C5—C6—C7	118.54 (15)	C13—C14—C15	120.16 (14)
C5—C6—H6	120.7	C16—C15—C14	120.57 (15)
C7—C6—H6	120.7	C16—C15—H15	119.7
C6—C7—C2	119.89 (15)	C14—C15—H15	119.7
C6—C7—C8	128.61 (15)	C15—C16—C11	121.01 (14)
C2—C7—C8	111.49 (14)	C15—C16—H16	119.5
C7—C8—C9	103.55 (13)	C11—C16—H16	119.5
C7—C8—H8A	111.1	O2—C17—H17	109.5
C9—C8—H8A	111.1	O2—C17—H17B	109.5
C7—C8—H8B	111.1	H17—C17—H17B	109.5
C9—C8—H8B	111.1	O2—C17—H17C	109.5
H8A—C8—H8B	109.0	H17—C17—H17C	109.5
C10—C9—C1	120.30 (14)	H17B—C17—H17C	109.5
			
O1—C1—C2—C3	0.6 (3)	C2—C1—C9—C8	0.59 (18)
C9—C1—C2—C3	179.53 (16)	C7—C8—C9—C10	178.56 (17)
O1—C1—C2—C7	−179.84 (16)	C7—C8—C9—C1	−0.04 (17)
C9—C1—C2—C7	−0.96 (18)	C1—C9—C10—C11	174.72 (16)
C7—C2—C3—C4	0.3 (3)	C8—C9—C10—C11	−3.7 (3)
C1—C2—C3—C4	179.78 (16)	C9—C10—C11—C12	−177.64 (17)
C2—C3—C4—C5	−0.5 (3)	C9—C10—C11—C16	0.0 (3)
C3—C4—C5—C6	0.4 (3)	C16—C11—C12—C13	−1.8 (2)
C4—C5—C6—C7	−0.1 (3)	C10—C11—C12—C13	176.01 (15)
C5—C6—C7—C2	−0.1 (2)	C11—C12—C13—C14	0.3 (3)
C5—C6—C7—C8	179.26 (16)	C17—O2—C14—C13	−0.6 (2)
C3—C2—C7—C6	0.0 (3)	C17—O2—C14—C15	179.49 (15)
C1—C2—C7—C6	−179.54 (15)	C12—C13—C14—O2	−178.74 (15)
C3—C2—C7—C8	−179.47 (15)	C12—C13—C14—C15	1.1 (2)
C1—C2—C7—C8	0.97 (19)	O2—C14—C15—C16	178.80 (15)
C6—C7—C8—C9	180.00 (16)	C13—C14—C15—C16	−1.1 (3)
C2—C7—C8—C9	−0.57 (18)	C14—C15—C16—C11	−0.4 (3)
O1—C1—C9—C10	0.7 (3)	C12—C11—C16—C15	1.8 (2)
C2—C1—C9—C10	−178.19 (15)	C10—C11—C16—C15	−175.80 (16)
O1—C1—C9—C8	179.47 (16)		

### Hydrogen-bond geometry (Å, º)

**Table:**

*D*—H···*A*	*D*—H	H···*A*	*D*···*A*	*D*—H···*A*
C13—H13···O1^i^	0.95	2.58	3.5327 (19)	175

Symmetry code: (i) −*x*+1, *y*−1/2, −*z*+1/2.
